# Exploring Determinants of Health-Related Quality of Life in Emerging Adults with Type 1 Diabetes Mellitus: A Cross-Sectional Analysis

**DOI:** 10.3390/nu16132059

**Published:** 2024-06-28

**Authors:** María-Ángeles Núñez-Baila, Anjhara Gómez-Aragón, Armando-Manuel Marques-Silva, José Rafael González-López

**Affiliations:** 1 Nursing Department, Faculty of Nursing, Physiotherapy and Podiatry, Universidad de Sevilla, 41009 Seville, Spain; mnbaila@us.es (M.-Á.N.-B.); joserafael@us.es (J.R.G.-L.); 2 Instituto de Biomedicina de Sevilla, IBiS/Hospital Universitario Virgen del Rocío/CSIC/Universidad de Sevilla, 41013 Sevilla, Spain; 3 Department of Nursing, Escola Superior de Enfermagem de Coimbra, 3004-011 Coimbra, Portugal; armandos@esenfc.pt; 4 Unidade de Investigação em Ciências da Saúde: Enfermagem (UICISA: E), 3004-011 Coimbra, Portugal

**Keywords:** emerging adult, Health-Related Quality of Life, Mediterranean diet, sleep, Type 1 Diabetes Mellitus

## Abstract

(1) Background: Emerging adulthood, from the age of 18 to 29 years, is a crucial phase for individuals with Type 1 Diabetes Mellitus, as it affects their Health-Related Quality of Life. (2) Methods: This cross-sectional study analyzes the influence of the Mediterranean diet, Diabetes duration, Hemoglobin A1c, and sleep disorders on Health-Relate Quality of Life in Type 1 Diabetes Mellitus. In this study, conducted in Andalusia, Spain, 362 emerging adults with Type 1 Diabetes Mellitus completed the Oviedo Sleep Questionnaire, the Adaptation of Mediterranean Diet Adherence Screener, and the Vida con Diabetes Tipo 1 (ViDa1) Health-Related Quality of Life questionnaire between October 2021 and July 2022. Pearson correlation coefficients and a multiple regression analysis were conducted for each Health-Related Quality of Life in Type 1 Diabetes Mellitus dimension (Interference with Life, Well-being, Self-care, and Concern about the Condition) for overall sample and separately for males and females. (3) Results: Different and significant correlations are found among factors such as Age, Body Mass Index, Currently being a student, Hemoglobin A1c, Sleep satisfaction, Insomnia, Hypersomnolence, and Adherence to Mediterranean diet. Notably, Insomnia is a main predictor for Interference with Life, Well-being, and Concern about the Condition, especially for females. (4) Conclusions: Insomnia is the main predictor of Health-Related Quality of Life in Type 1 Diabetes Mellitus among Andalusian emerging adults with this condition. Consequently, a regular assessment of sleep and Health-Related Quality of Life from a gender perspective in this age group is crucial.

## 1. Introduction

Health-Related Quality of Life (HRQOL) is the subjective evaluation of the health state individuals perceive about their physical, psychological and cognitive, and social well-being [1]. This appraisal is particularly useful for people with chronic, non-communicable diseases, as increasing the HRQOL is a therapeutic objective per se [1]. Currently, chronic diseases are a public health problem and they are usually diagnosed during adulthood or old age. However, Type 1 Diabetes Mellitus (T1DM) is usually diagnosed during childhood or adolescence [2]. Early-onset chronic diseases, such as T1DM, implies a longer time living with the disease and, therefore, a greater likelihood of complications and worse perception of HRQOL.

A multitude of factors play a significant role in HRQOL, including physical and mental well-being, social support—emphasized by the presence of positive interpersonal relationships—socioeconomic factors, access to healthcare services, and environmental conditions. Notably, gender also impacts HRQOL, with females typically experiencing lower HRQOL with T1DM [3,4,5]. Furthermore, effective disease management, which encompasses treatment adherence and appropriate symptom control, is a crucial determinant of HRQOL [5].

In terms of diabetes management, the impact of T1DM on HRQOL is modulated by various factors, including the complex nature of T1DM treatment, attitudes, coping mechanisms, and the presence of acute complications (such as hypoglycemia and hyperglycemia), as well as chronic complications (including retinopathy, neuropathy, cardiovascular disease, periodontal disease, and sexual dysfunction) [6,7,8]. Achieving an optimal HRQOL in T1DM requires a comprehensive treatment approach that encompasses insulin therapy, carbohydrate counting as part of dietary management [9], regular physical exercise [10], self-monitoring, and effective diabetes education to handle everyday challenges and exceptional situations [11]. However, factors that impact the HRQOL in T1DM, such as different aspects of lifestyle, particularly sleep or nutrition patterns, are less studied [12].

During childhood and adolescence, T1DM management is assumed, to a greater or lesser extent, by the relatives or legal tutors [13]. Nonetheless, during emerging adulthood, a stage defined by Arnett as spanning from ages 18 to 29 years, it is key to assume responsibilities toward gaining greater independence [14]. Less parental control and the adoption of new study or work schedules result in alterations in previous routines, particularly in relation to meals and sleep [14]. Considering these factors, it is crucial to assess dietary habits and sleep patterns in relation to the HRQOL in T1DM within this age group. The importance of this issue is magnified by the globally recognized risk of a potential discontinuity in medical care during the transition from pediatric to adult healthcare services [15].

Current research does not offer a global prevalence of sleep disorders among individuals with T1DM, but cross-sectional studies have reported rates ranging between 31% to 41% among people with T1DM [16,17,18,19]. In the field of T1DM, the Pittsburgh Sleep Quality Index (PSQI) is commonly used to evaluate sleep quality, as evidenced by numerous studies [12,20,21,22]. However, investigations specifically targeting distinct sleep disorders remain relatively scarce [23,24]. A significant association was suggested between this condition and the prevalence of sleep disorders, indicating that individuals with T1DM are particularly susceptible to developing sleep disorders, including sleep deprivation [23]. The effects of these sleep disorders include decreased insulin sensitivity, disruptions in physical and cognitive functioning, memory impairment, and alterations in appetite [23,25]. These effects influence the capacity of decision making the following day, which in turn affects blood glucose levels [23]. Sleep disorders and poor sleep quality have been associated with worse blood glucose levels and low HRQOL of individuals with T1DM [26]. However, these associations have predominantly been examined in pediatric and adolescent age groups, with limited research focusing on later stages of life [23,27].

In relation to nutrition, food selection and meal elaboration are milestones toward achieving independence, especially when the emerging adult lives outside the family home [28]. In this stage, due to the lack of time and high cost of fresh products, there is a high consumption of ultra-processed and pre-cooked food [28]. Emerging adulthood is a life stage that potentially represents a critical juncture in the establishment of dietary habits [11]. The continuation of unhealthy dietary practices, in due course, could be correlated to the onset of nutritional imbalance and diabetes-related complications, ultimately impacting the HRQOL of T1DM [29]. While there are multiple healthy diets that could be investigated for their benefits, this study focuses on the MedDiet (Mediterranean diet), particularly within the context of Southern Spain, known for its MedDiet adherence [30]. The MedDiet is chosen for its well-documented correlation with improved HRQOL, particularly in regions where it is deeply ingrained in the culture and lifestyle [30]. Despite the proven benefits of the MedDiet and its cultural significance in Southern Spain, adherence among individuals with T1DM remains low, even though diabetes education provides a high level of nutrition literacy [31,32]. Emerging adulthood is characterized by changes in and the experimentation of lifestyle behaviors. In this transitional and experimental stage, where individuals also begin to acquire independent management of T1DM, greater blood glucose instability may occur, therefore affecting the HRQOL in T1DM [11]. This is why the HRQOL assessment of emerging adults with T1DM is crucial.

For decades, various instruments have been utilized to assess HRQOL in individuals with diabetes [33,34,35,36]. The Vida con Diabetes Tipo 1 (ViDa1) questionnaire stands out for its improvements over previously used instruments. It offers high internal consistency, updated item language, specific design for adults with T1DM, high sensitivity to changes in insulin treatment, and a simple structure that avoids hypothetical scenarios for participants. These features enable more accurate and reliable measurements of HRQOL in individuals with T1DM [37]. However, there is a low number of studies focusing on emerging adulthood evaluating the HRQOL in T1DM, with tools specifically designed for T1DM in relation to factors that can determine it [6].

Despite the intimate relationship between sleep, diet, and HRQOL, there is a dearth of research that simultaneously explores these complex relationships. Most studies concentrate on the impact of these behaviors on diabetes management efficacy or blood glucose levels, rather than directly examining their influence on HRQOL in T1DM [12,23]. Therefore, our research question investigates whether the MedDiet, sociodemographic variables (Currently being a student or Currently being employed), T1DM variables, and sleep disorders (specifically Insomnia and Hypersomnolence), along with Sleep satisfaction, are associated with dimensions of HRQOL in T1DM—including Interference with Life, Self-care, Well-being, and Concern about the Condition—among Andalusian emerging adults with T1DM. Additionally, to avoid gender-related confounding factors, separate analyses were conducted for males and females. Consequently, we hypothesize that HRQOL in emerging adults with T1DM is worse in females and is significantly associated with the MedDiet, Currently being employed, blood glucose levels, Diabetes duration, and the presence of sleep disorders, both Insomnia and Hypersomnolence. In this sense, the aim of the study was to analyze the association of the MedDiet, sociodemographic variables, T1DM variables (Diabetes duration and Hemoglobin A1c (HbA1c) levels), and sleep disorders (excluding comorbidities or diabetes-related complications), on the HRQOL in emerging adults with T1DM.

Lastly, the novelty of this research lies in its focus on emerging adulthood, a crucial transitional stage that is often overlooked in comparison to childhood or adolescence. Additionally, our study utilizes a specific updated tool to assess HRQOL in individuals with T1DM and evaluates two lifestyle factors—nutrition patterns and sleep—which are profoundly influenced by the pursuit of independence during this life stage. Moreover, the dietary pattern studied aligns with those commonly associated with the sample region, lending greater consistency and relevance to the results. Furthermore, our study investigates Insomnia and Hypersomnolence symptoms, providing additional depth to our understanding of sleep disturbances in this specific demographic. These aspects, along with a comprehensive sex-disaggregated analysis, contribute to the originality of our findings, which significantly differ from previous research.

## 2. Materials and Methods

### 2.1. Design and Study Setting and Sampling

This cross-sectional study is embedded within a more extensive research project focusing on the quality of life and lifestyles of emerging adults with T1DM. Therefore, there are additional ongoing investigations using the same dataset; however, there is no overlap in variables.

This study was conducted from October 2021 to July 2022 in Andalusia (Spain). Inclusion criteria required participants to be aged between 18 and 29 years, with at least one year of T1DM development, and living in Andalusia. Individuals, irrespective of the presence of conditions that could potentially serve as confounding factors for the HRQOL in T1DM, were not excluded from the study. The Andalusian Health System User Database reported 5991 people with T1DM in this age group. In order to ensure representativeness, a confidence interval of 95% and a margin of error of 5% were established, obtaining a target sample of 361 participants of the population.

Participant recruitment was intentional, both face-to-face and online, through diabetes associations in Andalusia. Information flyers were posted on bulletin boards and handed over in public universities across Andalusia and strategic places, such as stationery/copy shops, and in the diabetes unit at university hospitals. In addition, a dissemination and recruiting campaign was launched on social media (Instagram and Facebook).

A Google Forms questionnaire was elaborated, where questions of three scales included a research overview and the lead researcher’s contact. Participants needed to consent to anonymous data use to proceed. The survey, taking about 25 min, required all questions to be answered to prevent data loss.

### 2.2. Measures

Through the questionnaire, self-reportedly, sociodemographic data and study variables were collected. The dependent variable was the HRQOL in T1DM, and independent variables were sleep (Insomnia, Hypersomnolence, and Sleep satisfaction), Adherence to MedDiet, HbA1c, Diabetes duration, and sociodemographic characteristics. Regarding the value of HbA1c, according to the guidelines of the Plan Integral de Diabetes de Andalucía [38], it is collected at least once per year in Andalusia. Sometimes, it could be collected every three or six months, depending on the assessment and needs. For this reason, it is interpreted that the HbA1c value self-reported in this study is the most recent and less than one-year-old because the questionnaire asked about the last value.

The global questionnaire integrated three validated questionnaires: a questionnaire to measure HRQOL in adults with T1DM (ViDa1) [37], Adaptation of Mediterranean Diet Adherence Screener (MEDAS), and the Oviedo Sleep Questionnaire (OSQ). Validity and reliability were ensured at the instrument’s creation, with Cronbach’s α values reported in each study [37,39,40].

For the questionnaires, there were no specific cutoff points; however, the cutoff point suggested by the Junta de Andalucía was set at 8 points for MEDAS [41]. For OSQ, a classification suggested by Romero et al. [42] was used, which categorizes Sleep satisfaction as low (1–3), medium (4), or high (5–7). Insomnia is classified as mild (9–21), moderate (22–33), or severe (34–45), and Hypersomnolence is classified as mild (1–5), moderate (6–10), or severe (11–15). This classification was used in the description of the results in the present study. A detailed description of each instrument is provided in Supplementary Material S1.

### 2.3. Bias

Participation was voluntary and fostered by intentional recruitment, enabling self-selection or voluntary bias, where participants may have any condition besides T1DM, which encouraged them to collaborate.

### 2.4. Data Analysis and Statistical Methods

Data were described in accordance to Strengthening Reporting of Observational Studies in Epidemiology (STROBE). The statistical analysis was performed with the software SPSS v26 (IBM). Through the Levene’s test for homogeneity of variance, using sex-disaggregated data and age intervals (18–24 and 25–29 years old), a homogeneous sample was observed. The sample size was large, thus, in adherence to the guidelines of the Central Limit Theorem [43] and verification through graphical analysis that the distribution is not excessively skewed or flat, the normality of the sample was assumed and parametric tests were applied. A descriptive analysis of sociodemographic data and a Pearson correlational analysis were conducted to determine the correlation among variables (Biological sex, Currently being a student, and Currently being employed are treated as dummy variables) and determine which ones will be included in the regression analysis.

In the multivariable analysis using forward multiple linear regression, we calculated the minimum sample size as N ≥ 50 + 8 m, choosing for the largest predictor count (6), thus requiring at least 98 participants [44]. Although we did not calculate the sample size for the regression model based on a power analysis, the table in the study by Green S. [44], which provides sample sizes derived from Cohen’s power analysis for 0.80 and α = 0.05, suggests our sample could be adequate for identifying both large and medium effects. Homoscedasticity for each independent variable was tested in each scatter chart. Independence in the residuals was assessed by the Durbin–Watson test, with values in the range of 1.5–2.5. The difference between the observed and expected values was ascertained using the Grubb’s outlier test, showing values of ±3 SD. All variables had a linear correlation instead of curvilinear. Finally, the absence of multicollinearity was also tested. Four models of multiple regression are presented, one per each dimension of the ViDa1 questionnaire, which served as the dependent variables. The selection of independent variables for each model was based on their statistically significant correlations with the dependent variable (each HRQOL dimension), as determined through Pearson correlation analysis. Regarding multicollinearity, we assessed the relationships among the predictors and found a strong relationship between Age and HbA1c. However, given the homogeneous nature of our sample, this strong relationship did not preclude our analysis. We acknowledge this limitation and recognize that it may affect the interpretation of our results. Furthermore, we applied the Bonferroni correction for multiple testing to minimize the risk of type I errors. We divided the significance level by four in our four regression models. This method adjusts the significance level to account for multiple comparisons [45]. Additionally, we conducted each detailed analysis (descriptive, correlational, and multiple linear regression) disaggregated by Biological sex. Similarly, parametric statistics were applied to these analyses, assuming homogeneity as verified for the entire sample, given the large sample sizes of each subset (118 males and 244 females).

### 2.5. Ethical Considerations

This study adheres to the Declaration of Helsinki and was approved by the Research Ethics Committee of the university hospitals Virgen Macarena and Virgen del Rocío in Seville, Spain, in their act of 11 February 2020, with the code 2150-M1-22. Participants were informed that their involvement was voluntary, anonymous, and solely for scientific use. Before starting the form, participants had to consent to the anonymous processing and sharing of their data. The questionnaire did not ask for personal identities and used provincial-level analyses with locality data to protect privacy.

## 3. Results

### 3.1. Characteristics of the Sample

In the study, 525 individuals participated; however, 163 were excluded for not meeting the inclusion criteria. The final sample included 362 participants. With respect to their sociodemographic variables (Table 1), participants had an average age of 22.77 ± 3.4 years, 67.4% were females, 68.5% had normal weight, and 81.5% were single. In relation to independent variables (Table 2), 52.1% of the sample described HbA1c > 53 mmol/mol and 73.5% of participants showed good Adherence to the MedDiet. Regarding sleep disorders, almost 40% (38.4%) of participants suffered from insomnia symptoms at a moderate or severe level. Similarly, almost 50% (49.7%) suffered moderate to severe hypersomnolence symptoms. Additionally, half of the emerging adults with T1DM reported sleep dissatisfaction ranging from very unsatisfied to indifferent. For a detailed analysis of the main variable (the HRQOL in T1DM), please refer to the study by Núñez-Baila, et al. [46].

### 3.2. Correlations between Sociodemographic Data, Diabetes (HbA1c and Diabetes Duration), MedDiet, Sleep, and HRQOL in T1DM in the Vida1 Questionnaire

The Pearson correlation analysis suggests that MedDiet correlates with the dimensions Self-care and Well-being. Sleep (Sleep satisfaction, Insomnia, and Hypersomnolence) has a very significant correlation with all HRQOL in T1DM dimensions. HbA1c correlates with all dimensions of HRQOL in T1DM, except with Interference with Life. Currently being a student correlates with Concern about the Condition. Notably, Diabetes duration and Currently being employed were the only factors that did not correlate with any HRQOL in T1DM dimensions. Table 3 contains significant Pearson correlations for each dimension of HRQOL in T1DM.

Biological sex correlates with up to three HRQOL in T1DM dimensions in the overall sample; sex-disaggregated analyses were conducted to enhance the understanding of these correlations. It has been observed that Hypersomnolence is associated with all HRQOL in T1DM dimensions, for both males and females. Meanwhile, Sleep satisfaction and Insomnia correlate with all dimensions for females; however, for males, no correlation exists between these variables and the dimension Concern about the Condition, nor for Insomnia with the Self-care dimension.

Age and BMI are shown to be predictors of Interference with Life and Well-being, respectively, and exclusively for females. Currently being a student is a predictor of Concern about the Condition only for males. Both HbA1c levels and Adherence to MedDiet also exhibit distinct correlations for males and females. Lastly, Diabetes duration and Currently being employed do not correlate with any dimension for either males or females. Table 4 contains a sex-disaggregated correlational analysis.

### 3.3. Multiple Regression Analysis Predicting HRQOL in T1DM Dimensions: Interference with Life, Self-Care, Well-Being, and Concern about the Condition

In the forward regression analysis, all potential variables were correlated with each dimension of HRQOL in T1DM. Variables with significant correlations were then used in the stepwise multiple regression analysis. Table 5 illustrates how the multiple regression analysis reveals predictors of each dimension of HRQOL in T1DM.

Regarding Interference with Life, Insomnia was the main predictor of this dimension. Insomnia explains the difficulty in diabetes management, affecting Interference with Life with an explanatory capacity of up to 12.2%. Additionally, with less explanatory power, there are other predictors, such as Hypersomnolence and Age, with explanatory capacities of 1.9% and 2%, respectively. The HbA1c value is the main predictor, with an explanatory capacity up to 11% of the Self-care dimension. Sleep satisfaction and Adherence to MedDiet are another two identified predictors with less explanatory power: 2.7% and 2%, respectively.

Again, Insomnia is the main predictor, with an explanatory power up to 40.4% for the Well-being dimension and diabetes management implied in this dimension. Though showing less explanatory power, the dimension of Well-being has the largest number of other predictors: Adherence to MedDiet, BMI, Hypersomnolence, HbA1c, Sleep satisfaction, and Biological sex. All of these variables are described in Table 5.

Finally, for the dimension Concern about the Condition, there are only two predictors: Insomnia as the main predictor, with an explanatory power of 6.1%, followed by Currently being a student, with an explanatory power of 1.4%.

After the Bonferroni correction for multiple testing, all predictors would have remained significant, except for HbA1c and Biological sex as predictors of Concern about the Condition, and Sleep satisfaction and Biological sex as predictors of Well-being. This is because its level of significance is below 0.05, but above 0.0125.

As with the correlations, multiple regression analyses disaggregated by Biological sex were conducted separately for males and females. While Hypersomnolence and Sleep satisfaction are unique predictors for males, Age and BMI are unique predictors for females. Other predictors, such as Adherence to MedDiet, HbA1c, and Insomnia, are present in both males and females, but across different dimensions. Notably, Insomnia is a predictor of the Well-being dimension, with an explanatory power of 32.8% for males and 38.6% for females. Table 6 concisely summarizes the predictors for each dimension of HRQOL in T1DM for males and females, with the explanatory power percentage listed under each predictor. Detailed sex-disaggregated regression analyses can be found in Tables S1 and S2 in Supplementary Material S2.

After the Bonferroni correction for multiple testing, all predictors remained significant, except Adherence to MedDiet and Currently being a student as predictors of Concern about the Condition for males and Insomnia as a predictor of Self-care for females. This is because its level of significance is below 0.05, but above 0.0125.

## 4. Discussion

It has been consistently observed that Insomnia is the main predictor in three of the four dimensions of the HRQOL of emerging adults with T1DM, particularly for females. Only in the dimension of Self-care, the main predictor is HbA1c for the overall sample, as well as for both males and females. Well-being is the dimension with a larger number of predictors, which suggests that attaining a high degree of well-being is much more complex. HbA1c as a predictor of the dimension Concern about the Condition can be considered natural and expected; however, Insomnia and Currently being a student are stronger predictors for this dimension. In the sex-disaggregated analysis, HbA1c disappears as a predictor of this dimension for both males and females. Adherence to MedDiet is only related with two dimensions: Self-care and Well-being. Adherence to MedDiet, as a predictor, ranks second for the Well-being dimension and third for the Self-care dimension for the overall sample.

In general, people with T1DM may feel their HRQOL threatened, especially when comparing it with the HRQOL of people without diabetes. Compared HRQOL differs according to age group, in pediatric ages. The study by Konstantaki et al. [47] does not establish any difference at the HRQOL level between children with and without diabetes. However, the study DAWN2 [48] points out a better the HRQOL in T1DM in later life stages (25–29 years old). On the contrary, in our study, increasing Age correlates with a greater Interference with Life, which suggests the detriment of the HRQOL in T1DM. This discrepancy could be explained by the fact that age increases the number of social, academic, and work responsibilities. The complexity of these responsibilities may interfere with daily life activity, if there are deficiencies in the development of adaptive strategies that allow individuals to meet these demands [48]. Specifically, our sex-disaggregated analysis suggests that the development of responsibilities could differ between males and females, as age remains a predictor only for females in Interference with Life.

During emerging adulthood, the probability of avoiding an insulin dose is greater [49], thus worsening the HbA1c values, which could explain the fact that 52.1% of our participants had an HbA1c above 53 mmol/mol, which is considered poor management of diabetes [49]. Additionally, it should be noted that Currently being a student predicts a lower concern about T1DM. This may be due to academic priorities taking precedence over diabetes self-care, thereby contributing to these results. Different studies on adults with T1DM [6,11,50] find no relation between HbA1c levels and the HRQOL in T1DM. By contrast, studies including adolescents with T1DM show a close relation between blood glucose levels and HRQOL, in line with our results [51,52]. Likewise, according to the results of the studies by Kent et al. and Stahl-Pehe et al. [6,52], there is no correlation between the Diabetes duration and the HRQOL in T1DM.

With respect to sleep, emerging adulthood is a life stage sensible to experiment sleep disorders, and almost 70% of university students declare themselves as ‘bad’ sleepers for sleeping less hours than the recommended time [53]. Some studies highlight that poor sleep quality does not predict nor correlate with the HRQOL of adolescents with or without T1DM [54,55]. In contrast, the review conducted by Perfect et al. [23] concluded that sleep disorders are more common in people with T1DM than in people not having this condition, which may explain the percentage of participants in this study that suffered from symptoms of sleep disorders. It is imperative to acknowledge that a significant proportion of our study population (38.4% for Insomnia and 49.7% for Hypersomnolence) exhibited moderate to severe insomnia or hypersomnolence symptoms, according to the suggested classification by Romero et al. [42]. Moreover, Insomnia is described as the main predictor of three out of four HRQOL in T1DM dimensions. This aligns with the results of the studies by Ichikawa et al. [27] and Griggs et al. [56], which include emerging adults with T1DM and observe how poor sleep affects both blood glucose levels and the HRQOL in T1DM. Additionally, being female is a demographic risk factor for insomnia [57]. Accordingly, our results indicate that Insomnia is the main predictor of HRQOL of females with T1DM, in contrast to males. In turn, poor sleep quality is associated with an impaired cognitive function, greater glycemic variability, HbA1c > 64 mmol/mol, and worse diabetes self-management [27,56]. The differences found between adolescent and emerging adult populations suggest that, in order to relate sleep to HRQOL in people with T1DM, the age group is relevant.

Regarding nutrition, the American Heart Association includes the MedDiet in their recommendations because it is associated with multiple health benefits and the prevention of cardiovascular diseases in people with diabetes [58]. Greater adherence to this nutrition pattern correlates with better blood glucose levels in children and adolescents with T1DM [58]. Furthermore, there is also a relation between adherence to MedDiet and better HRQOL in children and adolescents [59]. However, in later ages, such as emerging adulthood, adherence to MedDiet is not high and there is a progressive worsening of nutrition habits [60]. In this sense, the DAWN2 study [48] reveals a lower adherence to healthy diets in emerging adults with diabetes in comparison with later ages. Despite these data, this study observed that 73.5% of emerging adults showed adherence to MedDiet.

Research conducted by Mozzillo et al. [32] on Italian adolescents with T1DM did not find any evidence supporting the influence of the MedDiet on the HRQOL in T1DM. This result is attributed to the provision of comprehensive nutritional education following diagnosis. In contrast to Mozillo’s findings [32], our study aligns with the research conducted in Spain by Granado-Casas et al. [31], which involved an adult population with T1DM. This study revealed a significant correlation between moderate–high adherence to MedDiet and HRQOL of individuals with T1DM. At this point, except for the two aforementioned studies and this one, no other studies could be found evaluating the relation between the HRQOL in people with T1DM and adherence to MedDiet, though there is a vast amount of research on people with Type 2 Diabetes Mellitus, which provides evidence of the association between the MedDiet and HRQOL in individuals with diabetes [61].

### 4.1. Strengths and Limitations of This Study

This study evaluated HRQOL of individuals with T1DM during emerging adulthood using the ViDa1 questionnaire, a tool designed for assessing the HRQOL specifically associated with T1DM in adults. This is important because of the lack of specific tools for certain groups [6,51]. For instance, the PedsQL 3.2 questionnaire is only for children with T1DM and cannot be used for emerging adults older than 25 years old [62]. Our research investigated how HRQOL in emerging adults with T1DM is linked to two previously unexamined factors in this demographic: sleep disorders and adherence to MedDiet. Moreover, the utilization of a dietary patterns approach, as opposed to focusing solely on food intake or specific nutrients, is a notable strength of this study. Dietary patterns encompass the entirety of an individual’s diet over an extended period, providing a comprehensive understanding of nutritional habits. This holistic approach contrasts with reductionist methods that may isolate individual nutrients consumed over short periods [63]. This distinction is particularly pertinent in chronic disease research, where dietary choices established over time directly influence the development and progression of such conditions and their complications [64]. Therefore, adopting a long-term total diet approach is deemed most appropriate for investigating chronic diseases [65].

The main strength of this study lies in exploring an age group hardly researched to date. The results for the sociodemographic characteristics, blood glucose levels, Sleep satisfaction, Insomnia and Hypersomnolence symptoms, Adherence to MedDiet, and their relationship with the HRQOL of emerging adults with T1DM enrich the corpus of knowledge on the relationships little explored to date and, sometimes, contradictory in this population. Though recruitment was intentional, the sample used was representative and it had a significant size, which brings solidity to the study.

While the study acknowledges the inherent limitations associated with self-reported sociodemographic and clinical data, which are susceptible to memory biases, it is crucial to consider the countervailing factor of participant expertise. Individuals with T1DM often possess a deep understanding of the disease, effectively becoming ‘expert patients’ [66]. This profound knowledge base can enhance the credibility of their self-reported information, especially clinical data, potentially mitigating the impact of memory biases. The absence of data on socioeconomic status and ethnicity restricts the ability to generalize findings. Additionally, the analysis accounted for gender as a confounding factor by conducting separate comprehensive analyses for males and females, but did not consider potential confounding factors, such as comorbidities, diabetes-related complications, physical activity, exercise, and pain that could influence on the HRQOL of emerging adults with T1DM.

Furthermore, participants’ diabetes treatment data and diabetes devices were not considered in this research. Regarding glucose level measurement, although the Flash Glucose Monitor is currently funded by Spain and Andalusia for those with T1DM, its use is not mandatory, and when the study began, it was only available for children and adolescents. Considering that diabetes management technology can significantly influence outcomes and HRQOL for individuals with diabetes, the absence of these data in the adjusted models presents a notable limitation. Given the proliferation of diverse diabetes management technologies, future research endeavors should aim to comprehensively investigate the varying impacts of these technologies on HRQOL in individuals with T1DM.

Although we calculated the sample size based on a randomized study approach, our study used intentional sampling. While our sample size for the multiple regression model may be adequate, medium and large effects [44], in the field of health sciences, detecting subtle, small-scale effects can have significant clinical and public health implications. In this regard, there are criteria that allow for a smaller number of participants to be deemed an acceptable sample, further emphasizing the importance of our study [67]. Finally, despite regression analysis identifying potential predictor of HRQOL of emerging adults with T1DM, the cross-sectional design limits our ability to infer causality between variables.

The findings from this study offer a basis for future longitudinal research to explore causal relationships among factors affecting HRQOL of individuals with T1DM, including diabetes technology use. Investigating additional lifestyle factors, such as physical activity, exercise, substance use, and sexual behavior in emerging adults with T1DM, including them also as part of the investigation process, could further enhance this research field.

### 4.2. Study Implications for Practice

To improve the HRQOL of emerging adults with T1DM, all healthcare professionals, specifically diabetes nurse educators, should take into account that emerging adulthood can be a challenging stage to promote health. This population typically has a high level of health and may be unaware of the need to improve it. Regarding nutrition, it is crucial that nursing consultations go beyond carbohydrate counting and insulin adjustments.

Considering the hectic lifestyle typical in emerging adulthood, where nighttime leisure activities are commonplace and balancing studies with work is usually required, both scenarios can impact sleep patterns. Hence, it is crucial to recognize that, although it may initially appear that all our participants exhibit some degree of Insomnia or Hypersomnolence, this is not necessarily the case. This is because we adhered to the classification outlined by Romero et al. [42]. However, healthcare professionals should also consider gender differences due to the persistence of Insomnia as a predictor for females. Furthermore, the National Sleep Foundation has recognized that, although the cutoff point for assessing sleep efficiency is 85%, there exists a gray zone between 64% and 85% among emerging adults aged 18 to 25 years [68]. Within this range, it is not entirely clear whether individuals achieve optimal sleep efficiency [68]. Consequently, experiencing mild symptoms within this sample may be anticipated. It is important for healthcare professionals to understand that this is a special age group and the implications it holds for addressing sleep issues in clinical practice.

Therefore, nurses can play a pivotal role in developing tailored interventions to promote healthy dietary habits and enhance sleep health among emerging adults with T1DM, in line with the recommendations of the American Diabetes Association [69]. Similarly, regular health examinations can offer the opportunity to evaluate and enhance the HRQOL of emerging adults with T1DM across various aspects of their lifestyle. Consequently, healthcare professionals can adopt a comprehensive and holistic view of the health of emerging adults with T1DM.

## 5. Conclusions

Among the factors identified as HRQOL predictors in emerging adults with T1DM are HbA1c, BMI, Age, Currently being a student, Adherence to MedDiet, Insomnia, Hypersomnolence, and Sleep satisfaction. Insomnia stands out as the main predictor of HRQOL specific to T1DM in three of the four HRQOL dimensions (Interference with Life, Well-being, and Concern about the Condition) for emerging adults with T1DM. This is particularly evident in females, where Insomnia remains a primary predictor in these dimensions. In contrast, for males, Hypersomnolence replaces Insomnia in Interference with Life and Concern about the Condition, albeit with a lower explanatory power. Insomnia has even a greater explanatory power for HRQOL of individuals with T1DM than blood glucose levels measured through HbA1c, or even Adherence to MedDiet. Therefore, this study highlights the singularity of Insomnia as the predictor that can better explain HRQOL of individuals with T1DM during emerging adulthood.

## Figures and Tables

**Table 1:** Participant demographic profile. Participants (n = 362).

	Total Sample (n = 362)			Male (n = 118)			Female (n = 244)		
Variables	Mean ± SD/Range	n	%	Mean ± SD/Range	n	%	Mean ± SD/Range	n	%
Age Range: 18–29 years	22.77 ± 3.4			22.36 ± 3.41			22.98 ± 3.38		
18–24		244	67.4%		86	72.9%		158	64.8%
25–29		118	32.6%		32	27.1%		86	35.2%
Body Mass Index ^1^	24 ± 4.3 Range: 17.15–48.68			23.65 ± 3.10 Range: 18.10–35.27			24.20 ± 4.75 Range: 17.15–48.68		
Low weight		12	3.3%		1	0.8%		11	4.5%
Normal weight		248	68.5%		86	72.9%		162	66.4%
Pre-obesity or overweight		77	21.3%		27	22.9%		50	20.5%
Obesity class 1		13	3.6%		3	2.5%		10	4.1%
Obesity class 2		9	2.9%		1	0.8%		8	3.3%
Obesity class 3		3	0.8%		-	-		3	1.2%
Marital status									
Single		295	81.5%		92	78%		202	82.8%
Domestic partnership		62	17.1%		24	20.3%		39	16%
Married		5	1.4%		2	1.7%		3	1.2%
Andalusia: geographic dispersion									
Almeria		22	6.1%		6	5.1%		16	6.6%
Cadiz		38	10.5%		7	5.9%		31	12.7%
Cordoba		32	8.8%		10	8.5%		22	9%
Granada		53	14.6%		18	15.3%		35	14.3%
Huelva		16	4.4%		7	5.9%		9	3.7%
Jaen		21	5.8%		9	7.6%		12	4.9%
Malaga		61	16.9%		14	11.9%		47	19.3%
Seville		119	32.9%		47	39.8%		72	29.5%
Educational attainment: highest level of education completed or pursued									
Primary education		4	1.1%		2	1.7%		2	0.8%
Compulsory Secondary Education		29	8.0%		13	11%		16	6.6%
High School		58	16.0%		18	15.3%		40	16.4%
Intermediate vocational training		29	8%		11	9.3%		18	7.4%
Higher vocational training		58	16%		28	23.7%		30	12.3%
University degree		128	35.4%		35	29.7%		93	38.1%
Master or postgraduate		53	14.6%		10	8.5%		43	17.6%
Doctoral degree		3	0.8%		1	0.8%		2	0.8%
Currently being a student									
No		100	27.6%		38	32.2%		62	25.4%
Yes		262	72.4%		80	67.8%		182	74.6%
Employment status									
Does not work		178	49.2%		58	49.2%		120	49.2%
Full-time work		86	23.8%		33	28%		53	21.7%
Part-time work		37	10.2%		10	8.5%		27	11.1%
Casual or temporary work		50	13.8%		12	10.2%		38	15.6%
Self-employed work ^2^		10	2.8%		5	4.2%		5	2%
Currently being employed									
No		178	49.2%		58	49.2%		120	49.2%
Yes		183	50.6%		60	50.8%		123	50.4%

^1^ World Health Organization classification of adults according to Body Mass Index. ^2^ Self-employed work in the context of this study refers to individuals owning their own business, which often involves unique schedules and additional responsibilities not typically associated with other forms of employment. This distinction is particularly relevant in Spain, where self-employment can significantly impact stress levels and personal well-being due to due to the system of taxes and the complex bureaucracy, which is more challenging than in many other European countries.

**Table 2:** Independent variables of the study.

	Total Sample (n = 362)			Male (n = 118)			Female (n = 244)		
Variables	Mean ± SD/Range	n	%	Mean ± SD/Range	n	%	Mean ± SD/Range	n	%
Type 1 Diabetes Mellitus duration Range: 1–28 years	11.85 ± 6.79			11.85 ± 6.79			12.04 ± 6.88		
Hemoglobin A1c	Range: 27–130 mmol/mol			Range: 32–115 mmol/mol			Range: 27–130 mmol/mol		
Hemoglobin A1c ≤53 mmol/mol		172	47.1%		57	48.3%		115	47.1%
Hemoglobin A1c >53 mmol/mol		190	52.1%		61	51.7%		129	52.9%
Adherence to Mediterranean Diet									
Adherent (≥8)		266	73.5%		89	75.4%		177	72.5%
Non-adherent (<8)		96	26.5%		29	24.6%		67	27.5%
Insomnia									
Mild Insomnia		223	61.6%		95	80.5%		128	52.5%
Moderate Insomnia		111	30.7%		19	16.1%		92	37.7%
Severe Insomnia		28	7.7%		4	3.4%		24	9.8%
Hypersomnolence									
Mild Hypersomnolence		182	50.3%		76	64.4%		106	43.4%
Moderate Hypersomnolence		142	39.2%		34	28.8%		108	44.3%
Severe Hypersomnolence		38	10.5%		8	6.8%		30	12.3%
Sleep satisfaction									
Totally unsatisfied		23	6.4%		8	6.8%		15	6.1%
Very unsatisfied		40	11.0%		13	11%		27	11.1%
Unsatisfied		50	13.8%		9	7.6%		41	16.8%
On average		97	26.8%		30	25.4%		67	27.5%
Satisfied		88	24.3%		35	29.7%		53	21.7%
Very satisfied		50	13.8%		18	15.3%		32	13.1%
Totally satisfied		14	3.9%		5	4.2%		9	3.7%

**Table 3:** Pearson correlations among sociodemographic, diabetes, nutrition, and sleep variables, and Health-Related Quality of Life in Type 1 Diabetes Mellitus dimensions (Interference with Life, Self-care, Well-being, and Concern about the Condition).

Variables	Interference with Life	Self-Care	Well-Being	Concern about the Condition
Age	0.176 ***			
Biological sex	0.105 *		−0.264 ***	0.166 **
Body Mass Index			−0.168 **	
Hemoglobin A1c		−0.335 ***	−0.182 ***	0.124 *
Sleep satisfaction	−0.258 ***	0.203 ***	0.454 ***	−0.132 *
Insomnia	0.353 ***	−0.196 ***	−0.637 ***	0.252 ***
Hypersomnolence	0.332 ***	−0.180 ***	−0.520 ***	0.202 ***
Adherence to Mediterranean Diet		0.177 ***	0.229 ***	
Currently being a student				−0.105 *

* *p* < 0.05, ** *p* < 0.01, and *** *p* < 0.001.

**Table 4:** Sex-disaggregated Pearson correlations among diabetes, nutrition, and sleep variables, and Health-Related Quality of Life in Type 1 Diabetes Mellitus dimensions (Interference with Life, Self-care, Well-being, and Concern about the Condition).

	Variables	Interference with Life	Self-Care	Well-Being	Concern about the Condition
Age	Male				
	Female	0.192 **			
Body Mass Index	Male				
	Female			−0.178 **	
Hemoglobin A1c	Male		−0.284 **		
	Female		−0.360 ***	−0.237 ***	0.131 *
Sleep satisfaction	Male	−0.289 **	0.268 **	0.534 ***	
	Female	−0.235 ***	0.166 **	0.413 ***	−0.159 *
Insomnia	Male	0.269 **		−0.578 ***	
	Female	0.369 ***	−0.193 **	−0.623 ***	0.237 ***
Hypersomnolence	Male	0.432 ***	−0.228 *	−0.577 ***	0.237 **
	Female	0.271 ***	−0.147 *	−0.458 ***	0.147 *
Adherence to Mediterranean Diet	Male				−0.229 *
	Female		0.198 **	0.281 ***	
Currently being a student	Male				−0.217 *
	Female				

* *p* < 0.05, ** *p* < 0.01, and *** *p* < 0.001.

**Table 5:** Multiple regression analysis of Health-Related Quality of Life in Type 1 Diabetes Mellitus dimensions (Interference with Life, Self-care, Well-being, and Concern about the Condition).

	**Interference with Life**	
**Predictors**	**Standardized** **Coefficients Beta**	**Adjusted R^2^**
Insomnia	0.206 **	0.122
Age	0.166 ***	0.142
Hypersomnolence	0.198 **	0.161
F (3,358) = 24.069; *p* < 0.05; R^2^ = 0.168		
	**Self-care**	
**Predictors**	**Standardized** **coefficients beta**	**Adjusted R^2^**
Hemoglobin A1c	−0.307 ***	0.110
Sleep satisfaction	0.157 **	0.137
Adherence to Mediterranean Diet	0.126 *	0.150
F (3,358) = 22.206; *p* < 0.05; R^2^ = 0.157		
	**Well-being**	
**Predictors**	**Standardized** **coefficients beta**	**Adjusted R^2^**
Insomnia	−0.441 ***	0.404
Adherence to Mediterranean Diet	0.134 ***	0.426
Body Mass Index	−0.115 **	0.441
Hypersomnolence	−0.150 **	0.453
Hemoglobin A1c	−0.110 **	0.465
Sleep satisfaction	0.113 *	0.470
Biological sex	−0.097 *	0.477
F (7,354) = 48.050; *p* < 0.05; R^2^ = 0.487		
	**Concern about the Condition**	
**Predictors**	**Standardized** **coefficients beta**	**Adjusted R^2^**
Insomnia	0.223 ***	0.061
Currently being a student	−0.145 **	0.075
Hemoglobin A1c	0.122 *	0.086
Biological sex	0.112 *	0.095
F (4,357) = 10.476; *p* < 0.05; R^2^ = 0.105		

* *p* < 0.05, ** *p* < 0.01, and *** *p* < 0.001.

**Table 6:** Summary of predictors from sex-disaggregated multiple regression analysis of Health-Related Quality of Life in Type 1 Diabetes Mellitus dimensions (Interference with Life, Self-Care, Well-Being, and Concern about the Condition).

Interference with Life		Self-Care		Well-Being		Concern about the Condition	
Male	Female	Male	Female	Male	Female	Male	Female
Hypersomno- Lence (17.9%)	Insomnia (13.2%) Age (2.7%)	HbA1c (7.3%) Sleep Satisfaction (5.8%)	HbA1c (12.6%) MedDiet (3%)	Insomnia (32.8%) Sleep Satisfaction (8.8%) Hypersomno- lence (4.2%)	Insomnia (38.6%) MedDiet (2.9%) BMI (2.5%) HbA1c (2.0%)	Hypersomno-lence (4.8%)	Insomnia (5.2%)

Abbreviations: HbA1c: Hemoglobin A1c; MedDiet: Adherence to Mediterranean Diet; BMI: Body Mass Index.

## Data Availability

The data presented in this study are available upon reasonable request. The data are available from María-Ángeles Núñez-Baila (email: mnbaila@us.es).

## Supplementary Materials

The following supporting information can be downloaded at: https://www.mdpi.com/article/10.3390/nu16132059/s1, Supplementary Material S1: Detailed description of questionnaires utilized in the study. Supplementary Material S2: Table S1. Sex-disaggregated multiple regression analysis of Health-Related Quality of Life in Type 1 Diabetes Mellitus dimensions (Interference with Life, Self-care, Well-being, and Concern about the Condition). Analysis for males. Table S2. Sex-disaggregated multiple regression analysis of Health-Related Quality of Life in Type 1 Diabetes Mellitus dimensions (Interference with Life, Self-care, Well-being, and Concern about the Condition). Analysis for females. Ref. [70] is cited in Supplementary Materials.
