# Supplementary material for: Exploring Determinants of Health-Related Quality of Life in Emerging Adults with Type 1 Diabetes Mellitus: A Cross-Sectional Analysis
Source: Nutrients. 2024 Jun 28;16(13):2059. doi: 10.3390/nu16132059 (PMC11243684; doi:10.3390/nu16132059)
Supplement: Supplementary file 1 [file nutrients-16-02059-s001.zip › Supplementary material 1 Rev_.pdf]

Supplementary Material 1

Detailed description of questionnaires utilized in the study.

Vida con Diabetes tipo 1 (ViDa1) questionnaire™ to assess the Health-Related Quality of Life in Type 1 Diabetes Mellitus.

The ViDa1 questionnaire™ has 34 items and it is divided in 4 dimensions/subscales (Interference with Life, Self-care, Well-being, and Concern about the Condition) displaying a 5-point. Likert-type response scale (1 = strongly disagree; 2 = disagree; 3 = neither agree nor disagree; 4 = agree; 5 = strongly agree), obtaining total score (12-60; 11-55; 6-30 and 5-25 respectively) for each dimension. There were no specific cut points as this instrument measured a subjective construct (Cronbach's α = 0.71-0.86) [37].

Adaptation of Mediterranean Diet Adherence Screener (MEDAS) to assess the adherence to Mediterranean Diet.

MEDAS based on the version validated by Schröder (Cronbach's α = 0.61) [40]. In this research, the nutrition pattern was evaluated with a questionnaire of adherence to Mediterranean diet adapted by the Junta de Andalucía with 14 questions. Items had the minimum required scoring established in a single reply, therefore becoming a dichotomous reply. Replies closest to Mediterranean diet adherence scored one point, and those farther from Mediterranean diet adherence scored zero points. Scoring Interval was 0 to 14. There were no specific cut points in this instrument, but in the current adaptation of this questionnaire conducted by the Junta de Andalucía in the guidelines *Guía de Recomendaciones sobre Hábitos Saludables en Atención Primaria* [41], the following question: *"Do you drink wine? How often in a week?"*, which response *"Three or more per week"* scored one point, has been eliminated, and therefore, we eliminated too. Thus, the questionnaire consisted in 13 questions and the cut-off point suggested by the Junta de Andalucía was set in 8 points.

Oviedo Sleep Questionnaire (OSQ) to assess the Sleep satisfaction, insomnia and hypersomnolence symptoms.

The OSQ is a tool which provides diagnostic assistance to evaluate sleep disorders (Cronbach's α = 0.7667) [70]. The questionnaire has 15 items: Sleep satisfaction (1 item), Insomnia (9 items), and Hypersomnolence (3 items), which scores range 1-7, 9-45, and 3-15 points, respectively. Each item is assigned a value 1 a 5 (Likert scale), except item 1 which scores 1 to 7. In the subscale of Insomnia or Hypersomnolence, a higher score indicates greater severity. This is a validated scale for people with depression, bipolar disorder, and schizophrenia [70]. However, it has also been used in other groups, such as workers subjected to stressful situations [42]. Despite the aforementioned considerations, the appropriateness of this scale for this population lies in its conciseness and straight-forwardness, exemplified by inquiries such as, "How many days a week have you had difficulty staying asleep?" (possible responses: none, 1-2 days, 3 days, 4-5 days, or 6-7 days). This simplification makes it a particularly suitable instrument for younger individuals, who frequently have limited time available for comprehensive surveys.

Additionally, this scale possesses the capacity to assess the potential presence or absence of a sleep disorder, conditions that tend to be more prevalent in individuals with T1DM compared to those without T1DM [23]. This tool does not establish cut-off points, although depending on the degree of affectation, there is a suggested classification of mild, moderate and severe in the study by Romero et al. [42]. In this classification, the Sleep satisfaction could be low (1-3) medium (4) or high (5-7), regarding the Insomnia could be mild (9-21); moderate (22-33) and severe (34-45) and finally, about the Hypersomnolence could be mild (1-5) moderate (6-10) or severe (11-15). This classification was used in the description of the results in the present study.

References corresponding to the manuscript

23. Perfect, M.M. Sleep-Related Disorders in Patients with Type 1 Diabetes Mellitus: Current Insights. *Nat. Sci. Sleep* **2020**, *12*, 101–123. https://doi.org/10.2147/NSS.S152555.

37. Alvarado-Martel, D.; Ruiz Fernández, M.A.; Cuadrado Vigaray, M.; Carrillo, A.; Boronat, M.; Expósito Montesdeoca, A.; Nattero Chávez, L.; Pozuelo Sánchez, M.; López Quevedo, P.; Santana Suárez, A.D.; et al. Vida1: The Development and Validation of a New Questionnaire for Measuring Health-Related Quality of Life in Patients with Type 1 Diabetes. *Front. Psychol.* **2017**, *8*, 14. https://doi.org/10.3389/fpsyg.2017.00904.

40. Schröder, H.; Fitó, M.; Estruch, R.; Martínez-González, M.A.; Corella, D.; Salas-Salvadó, J.; Lamuela-Raventós, R.; Ros, E.; Salaverría, I.; Fiol, M.; et al. A Short Screener Is Valid for Assessing Mediterranean Diet Adherence among Older Spanish Men and Women. *J. Nutr*. **2011**, *141*, 1140–1145. https://doi.org/10.3945/jn.110.135566.

41. Maqueda, R.Á. (coordination). Recomendaciones Sobre Hábitos Saludables En Atención Primaria: Modelo de Atención Para Promover La Actividad Física y La Alimentación Equilibrada En El Sistema Sanitario Público de Andalucía; 2020. Consejería de Salud y Familias, Junta de Andalucía. Available online: https://www.juntadeandalucia.es/organismos/saludyconsumo/areas/salud-vida/adulta/paginas/consejo-dietetico.htm (accessed on 27 June 2024).

42. Romero Paredes, M.d.C.; Reinoso Barbero, L.; González Gómez, M.F.; Bandrés Moya, F. Estudio Del Sueño En Un Grupo de Trabajadores Del Mar Mediante La Aplicación Del Cuestionario de Oviedo Correspondencia. *Rev. La Asoc. Española Espec. En Med. Del Trab.* **2017**, *26*, 110–120.

70. Paz García-Portilla, M.; Alejandra Sáiz, P.; Díaz-Mesa, E.M.; Fonseca, E.; Arrojo, M.; Sierra, P.; Sarramea, F.; Sánchez, E.; Manuel Goikolea, J.; Balanzá, V.; et al. Psychometric Performance of the Oviedo Sleep Questionnaire in Patients with Severe Mental Disorder. *Rev. Psiquiatr. y Salud Ment. (Engl. Ed.)* **2009**, *2*, 169–177. https://doi.org/10.1016/s2173-5050(09)70049-4.
