# Supplementary material for: Exploring Determinants of Health-Related Quality of Life in Emerging Adults with Type 1 Diabetes Mellitus: A Cross-Sectional Analysis
Source: Nutrients. 2024 Jun 28;16(13):2059. doi: 10.3390/nu16132059 (PMC11243684; doi:10.3390/nu16132059)
Supplement: Supplementary file 1 [file nutrients-16-02059-s001.zip › Supplementary material 2 Rev_.pdf]

Supplementary Material 2

Table S1. Sex-disaggregated multiple regression analysis of Health-Related Quality of Life in Type 1 Diabetes Mellitus dimensions (Interference with Life, Self-care, Well-being, and Concern about the Condition). Analysis for males.

	Interference with Life	
Predictor	Standardized coefficients beta	Adjusted R^2
Hypersomnolence	0.432***	0.179
F (1,116) =26.586; p<0.001; R^2=0.186		
	**Self-care**	
Predictors	Standardized coefficients beta	Adjusted R^2
Hemoglobin A1c	-0.272**	0.073
Sleep satisfaction	0.254**	0.131
F (2,115) =9.791; p<0.001; R^2=0.145		
	**Well-being**	
Predictors	Standardized coefficients beta	Adjusted R^2
Insomnia	-0.201	0.328
Sleep satisfaction	0.330***	0.416
Hypersomnolence	-0.311**	0.458
F (3,114) =33.947; p<0.001; R^2=0.472		
	**Concern about the Condition**	
Predictors	Standardized coefficients beta	Adjusted R^2
Hypersomnolence	0.230*	0.048
Currently being a student	-0.200*	0.092
Adherence to Mediterranean Diet	-0.180*	0.117
F (3,114) =6.144; p<0.01; R^2=0.139		

*p<0.05, **p<0.01 and *** p<0.001

Table S2. Sex-disaggregated multiple regression analysis of Health-Related Quality of Life in Type 1 Diabetes Mellitus dimensions (Interference with Life, Self-care, Well-being, and Concern about the Condition). Analysis for females.

	Interference with Life	
Predictors	Standardized coefficients beta	Adjusted R^2
Insomnia	0.360***	0.132
Age	0.175**	0.159
F (2,241) =24.027; p<0.001; R^2=0.166		
	†Self-care	
Predictors	Standardized coefficients beta	Adjusted R^2
Hemoglobin A1c	-0.337***	0.126
Adherence to Mediterranean Diet	0.162**	0.156
Insomnia	-0.121*	0.167
F (3,240) =17.181; p<0.001; R^2=0.177		
	Well-being	
Predictors	Standardized coefficients beta	Adjusted R^2
Insomnia	-0.569***	0.386
Adherence to Mediterranean Diet	0.179***	0.415
Body Mass Index	-0.161***	0.440
Hemoglobin A1c	-0.148**	0.460
F (4,239) =52.709; p<0.001; R^2=0.469		
	Concern about the Condition	
Predictor	Standardized coefficients beta	Adjusted R^2
Insomnia	0.237***	0.052
F (1,242) =14.456; p<0.001; R^2=0.056		

*p<0.05, **p<0.01 and *** p<0.001

† The difference between the observed and expected values was ascertained using the Grubb's Outlier Test, showing values -3.505 ~ 2.468 for Self-care dimension.
